# Critical Connections: Diagnosis of Epstein-Barr Virus (EBV)-Positive Nasopharyngeal Carcinoma-Associated Hemophagocytic Lymphohistiocytosis

**DOI:** 10.7759/cureus.95456

**Published:** 2025-10-26

**Authors:** Seyed Khalafi, Jeffrey Tessier, Tiffany Lee

**Affiliations:** 1 Internal Medicine, University of Texas Southwestern Medical Center, Dallas, USA; 2 Internal Medicine/Infectious Diseases, University of Texas Southwestern Medical Center, Dallas, USA; 3 Internal Medicine/Hospital Medicine, University of Texas Southwestern Medical Center, Dallas, USA

**Keywords:** elevated ferritin level, epstein-barr virus (ebv), hemophagocytic lymphohistiocytosis (hlh), nasopharyngeal cancer (npc), rare differential

## Abstract

Hemophagocytic lymphohistiocytosis (HLH) is a rare and potentially fatal condition associated with Epstein-Barr virus (EBV) in children and adults. We report a case of a 65-year-old male with a history of EBV-positive nasopharyngeal carcinoma in remission presenting with fever, altered mental status, abdominal pain, and generalized weakness. Labs showed pancytopenia, hyponatremia, hypochloremia, acute-on-chronic kidney injury, and hypoalbuminemia. The patient was started on empiric broad-spectrum antimicrobials, intravenous crystalloids, and red blood cell transfusions. HLH was suspected, and laboratory evaluation demonstrated elevated EBV viral load, ferritin, and soluble interleukin-2 receptor levels. He met diagnostic criteria for HLH and was started on etoposide and methylprednisolone. However, due to rapid deterioration, the family requested hospice care, and the patient passed away four days later. This case highlights severe presentations of HLH and the importance of considering HLH in the differential diagnosis of patients with nasopharyngeal carcinoma presenting with undifferentiated multisystem syndromes.

## Introduction

Hemophagocytic lymphohistiocytosis (HLH) is a rare and potentially fatal syndrome that can manifest from primary (familial/hereditary) or secondary (non-familial/hereditary) causes. The most common causes of secondary HLH include infections, collagen diseases, or malignancies. Of the secondary causes, viruses are a major type of infection-induced HLH, with Epstein-Barr virus (EBV) being the most commonly implicated virus [1,2]. An increase in EBV viral load and inadequate T cell expansion leads to uncontrolled B-cell lymphoproliferative disorders. In addition, predisposed T-cell or natural killer (NK) cell dysfunction, whether genetic or environmental, will also result in sustained T-cell activation and unchecked and substantial cytokine release and macrophage activation. This dysregulation of cell-mediated immunity and overproduction of cytokines results in immune-mediated end-organ damage [3]. In this case report, we present a 65-year-old male with multiple hospitalizations who was subsequently found to have EBV-induced HLH.

## Case presentation

A 65-year-old Caucasian male with T2 N3 nasopharyngeal carcinoma (EBV-positive), status post concurrent gentamicin/cisplatin and radiation therapy, Parkinson’s disease, chronic kidney disease stage 3a, and pancytopenia initially presented with fever, altered mental status, abdominal pain, and generalized weakness. He had previously been admitted in the last month for similar symptoms and was found to have gallbladder distention without stones on abdominal ultrasound; he was treated with metronidazole and ceftriaxone with subsequent symptom improvement and discharged home. He subsequently developed worsening cognition, Parkinsonian symptoms, declining activities of daily living, incontinence, and anorexia with a 20-pound weight loss in the last month.

On presentation, he was febrile (38.4°C), tachycardic (110 beats/min), tachypneic (20 breaths/min), and hypotensive (99/43 mmHg with a mean arterial pressure of 62 mmHg) and had an oxygen saturation (SatO_2_) of 94% on room air. On the physical exam, he was somnolent, cachectic, alert, and oriented to name and place only and in acute distress. He had dry mucous membranes, poor dentition, and a resting right arm tremor.

He was started empirically on intravenous (IV) piperacillin/tazobactam, vancomycin, and crystalloid fluid resuscitation. A complete blood count panel revealed anemia with a hemoglobin of 4.1 g/dL, and he was given two units of packed red blood cells.

Admission labs showed hyponatremia, hypochloremia, acute-on-chronic kidney injury, and hypoalbuminemia (Table 1).

**Table 1:** Summary of laboratory studies (total of eight hospitalization days) HD: hospital day, WBC: white blood cells, HGB: hemoglobin, HCT: hematocrit, pCO₂: partial pressure of carbon dioxide, EBV: Epstein-Barr Virus, IL: interleukin

	HD 1	HD 3	HD 5	HD 7	Reference ranges
pCO₂ (mmHg)				33.7	39–55
WBC (x10³/L)	2.46	1.80	1.73	2.95	4.0–11.0
HGB (g/dL)	4.1	7.6	6.6	7.1	12.4–17.3
HCT (%)	12.7	21.8	20.2	22.1	37.0–50.0
Platelets (x10^9^/L)	114	90	53	40	150–450
Serum sodium (mmol/L)	127	127	139	144	135–145
Serum potassium (mmol/L)	4.7	4.2	3.6	3.5	3.5–5.1
Serum chloride (mmol/L)	96	97	110	118	98–107
Serum glucose (mg/dL)	73	67	86	172	74–106
Blood urea nitrogen (mg/dL)	46	54	37	42	6–23
Serum creatinine (mg/dL)	1.86	2.13	1.66	1.45	0.72–1.25
Alkaline phosphatase (Units/L)	69			56	40–150
Aspartate transferase (Units/L)	162			133	10–50
Alanine transferase (Units/L)	<6			9	10–50
Serum lactic acid (mmol/L)	1.7	1.1			0.7–2.1
Lactate dehydrogenase (Units/L)	2,971				125–220
Serum iron (mcg/dL)	18				37–158
TIBC (mcg/dL)	125				262–474
Percent saturation (%)	14				20–50
Ferritin (ng/mL)	11,304		43,270	55,905	22–275
Serum triglyceride (mg/dL)	122		316	453	<150
EBV viral load (IU/mL)		160,275		19,547	≤50
Soluble IL-2 receptor (pg/mL)	2,931.7		3,230.6		≤2.1
C-reactive protein (mg/L)		55.4			≤5.0
Serum kappa free light chain (mg/L)			233.21		3.30–19.40
Serum lambda free light chain (mg/L)			205.12		5.71–26.30
Beta globulin (g/dL)			0.22		0.30–0.59

Liver function testing showed elevated aspartate aminotransferase. The iron panel showed low iron, low total iron binding capacity, low percent saturation, and severely elevated ferritin of 11,304. Triglycerides, C-reactive protein, and lactate dehydrogenase levels were also elevated. Both urine and blood cultures had no growth.

Initial computed tomography (CT) of the abdomen/pelvis without IV contrast showed splenomegaly and no acute abnormalities. Additionally, a CT brain, lumbar spine, and chest without IV contrast and a transthoracic echocardiogram showed no acute abnormalities and an ejection fraction of 63%. Electroencephalography results showed generalized periodic discharges without seizures. Brain magnetic resonance imaging with and without contrast also showed no acute abnormalities. 

Based on the presentation with recurrent fever, progressive multiorgan dysfunction, and laboratory and imaging findings concerning HLH, soluble interleukin (IL)-2 receptor, triglycerides, NK cell activity, and flow cytometry were ordered (Table 1). His plasma quantitative EBV polymerase chain reaction (EBV viral load) was elevated, and IV acyclovir was initiated. A bone marrow biopsy was deferred as the clinical suspicion of HLH was confirmed through lab testing and imaging, and thus the patient was started on etoposide and methylprednisolone on hospital day 5. However, due to his continued clinical deterioration during his admission, the family decided to pursue comfort care measures only, and the patient passed away on hospital day 8. 

## Discussion

In the current study, we present a rare case of a patient with treated nasopharyngeal carcinoma (EBV-positive), presenting with concurrent fever, splenomegaly, pancytopenia, hypertriglyceridemia, very elevated ferritin, and elevated soluble IL-2 receptor. Taken together, this points towards a diagnosis of HLH. Our patient also manifested a rare presentation of HLH. The majority of published studies of HLH are derived from pediatric cases [4] while our patient was an adult male. Malignancy-associated HLH is most commonly seen in T or NK cell lymphoma or leukemia. Solid tumors least commonly account for 1.4% of HLH cases in the setting of diffuse metastatic disease with bone marrow infiltrates and aggressive histology [5]. However, there is no mention of any EBV-associated solid tumors. In addition, a review of the literature only reported 6 cases of nasopharyngeal carcinoma-associated HLH. 

Clinical manifestations of HLH include, but are not limited to, fever, splenomegaly, seizures, and mental status changes [2,6]. Due to many of the symptoms being observed in many other diseases, such as infection, malignancy, and autoimmune disorders, the diagnosis of HLH is commonly missed [6]. Diagnostic criteria have not been established for adults; however, our patient met 6/8 of the HLH-2004 diagnostic criteria from the Histiocyte Society (Table 2) [4,7].

**Table 2:** Histiocyte Society HLH-2004 diagnostic criteria Note: Adapted from references [4,7]. HLH: hemophagocytic lymphohistiocytosis

The diagnosis of HLH requires either one or two below to be fulfilled:
(1) A molecular diagnosis of HLH
(2) Diagnostic criteria of HLH fulfilled (five out of the eight criteria below)
Fever
Splenomegaly
Cytopenia (affecting ≥2 of 3 lineages in the peripheral blood):
Hemoglobin <90 g/L (in infants <4 weeks: hemoglobin <100 g/L)
Platelets <100 × 109/L
Neutrophils <1.0 × 109/L
Hypertriglyceridemia and/or hypofibrinogenemia:
Fasting triglycerides ≥3.0 mmol/L (i.e., ≥265 mg/dL)
Fibrinogen ≤1.5 g/L
Hemophagocytosis in bone marrow, spleen, or lymph nodes
Low or absent natural killer cell activity
Ferritin ≥500 mg/L
Soluble interleukin-2 receptor ≥2400 U/mL

Bone marrow biopsy, once considered the diagnostic gold standard for HLH, may be inconclusive due to possibly only revealing erythroid hyperplasia and causing clinicians to reject HLH [8]. This further justifies the need for more validated diagnostic criteria for adults, and new tests are being developed to adjust for the diagnosis of HLH in adults, one of which is the HScore. It consists of nine variables, which include clinical, cytological, and biological measurements, with the score ranging from 0 to 302. The score was noted to have a 93% sensitivity and 86% specificity for acquired HLH in adults [9,10]. Our patient had an HScore of 204 with a 90% chance of having a hemophagocytic lesion, further supporting our patient’s diagnosis of HLH. The mortality of HLH in adults ranges from 20.4 to 88%, further exemplifying the importance of early diagnosis and treatment [4]. While limiting factors of the study may have been that we were unable to obtain a bone marrow biopsy of the patient due to hemodynamic instability and obtaining serum fibrinogen or NK cell activity, we believe that his significantly high diagnostic score was sufficient to make a definitive diagnosis. 

**Figure 1:**
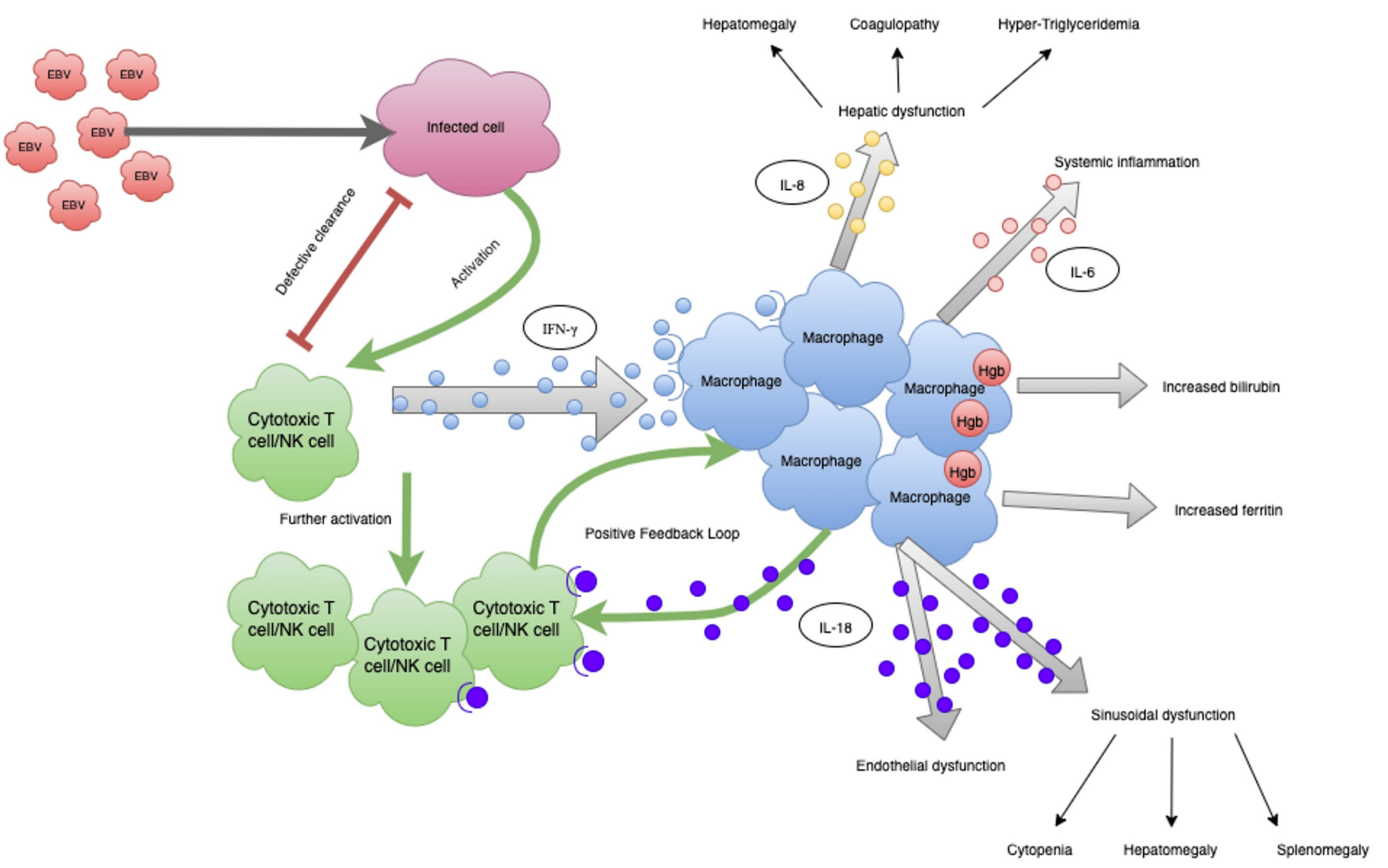
Schematic overview of the pathogenesis of HLH syndrome. HLH: hemophagocytic lymphohistiocytosis; EBV: Epstein-Barr virus; NK: natural killer; IL: interleukin; IFN: interferon; Hgb: hemoglobin Note: Adapted from reference [3].

The manifestations and lab findings seen in HLH can be further explained by the cytokine release and macrophage activation. The macrophages begin to phagocytize and break down heme into iron, resulting in elevated ferritin. In addition to extensive hemoglobin consumption, IL-18 release causes sinusoidal dysfunction with further cytopenia and hepatosplenomegaly. The hypermetabolic effects from the cytokine storm also contribute to the increased breakdown of lipid particles, causing hypertriglyceridemia and fever. This cytokine storm also produces a disseminated intravascular coagulopathy-like reaction, leading to overactivation of fibrinolysis and hypofibrinogenemia. Elevated soluble IL-2 receptors stem from the overly active T cells that express these receptors. In addition, the interferon gamma particles produced by macrophages cause a feedback loop further activating T cells and NK cells; however, over time, the NK cells show an exhausted phenotype, leading to decreased NK cell activity on testing [3] (Figure 1).

Adults with HLH have been observed to have a significant risk of mortality. Previous studies by Bichon et al. and Knaak et al. have shown a mortality rate of 57-60% regardless of etiology [11,12]. Patients with EBV-induced HLH have an overall survival rate of 41.1% at six months. In addition, a study by Yao et al. showed that platelets, C-reactive protein, creatinine, blood urea nitrogen, estimated glomerular filtration rate (eGFR), carbon dioxide levels, beta-2-microglobulin, procalcitonin, and soluble IL-2 receptor have the best ability to predict survival. Overall survival was significantly worse when procalcitonin was >1.8 ng/mL or when any two or more of soluble IL-2 receptor >18,000 pg/mL, procalcitonin >1.8 ng/mL, and estimated glomerular filtration rate (eGFR) <90 mL/min/1.73m² were met [13]. Our patient was noted to have a low eGFR and elevated soluble IL-2 receptor, suggesting that our patient was likely to have high HLH-related mortality. 

The recommended treatments for HLH are heterogeneous, with the most commonly suggested medications including a combination of corticosteroids ± chemotherapeutic options. The most common chemotherapeutic option was etoposide [14]. A study conducted by Bergsten et al. showed an HLH-associated mortality rate of 37% when started on a regimen of etoposide and corticosteroids. However, the regimen does not come without complications/sequelae, as etoposide is associated with secondary acute myeloid leukemia and hepatobiliary complications [15,16]. Other regimens that have been recommended include rituximab, emapalumab, or ruxolitinib [1]. An overall response rate of 69.2% and 64.7% was observed with ruxolitinib or emapalumab, respectively [17,18]. In addition, rituximab was shown to have a 26.7% complete remission rate and a 66.7% partial remission rate [19]. Importantly, etoposide only removes T-cells and NK cells from circulation and has no effect on reservoir B-cells where EBV is replicating, perhaps explaining relapsing or refractory HLH. It has been suggested that a combination of rituximab and etoposide be used, with stem cell transplantation as a salvage option [20]. It is also noted that patients who do not achieve a partial response by four weeks have a high mortality risk [21]. Acyclovir has been used to treat EBV infections, but it has not improved outcomes in patients with EBV-induced HLH [1], likely due to the lack of antiviral activity against the lysogenic phase of viral infection. Our patient was started on a combination of etoposide and methylprednisolone, as well as acyclovir for EBV viral load reduction; however, the treatment was discontinued two days later due to the family’s request for inpatient hospice. 

## Conclusions

In this case report, we present a rare and poor prognostic diagnosis of EBV-positive nasopharyngeal carcinoma-associated HLH. Our patient was noted during his admission to have a significantly elevated EBV viral load, which was determined to be the cause of his HLH. He was treated with etoposide, methylprednisolone, and acyclovir; however, due to the patient’s rapid deterioration, the family requested termination of treatment. Our case portrays the importance of early diagnosis of HLH and the vague signs and symptoms of EBV-induced HLH, as well as the management of this disease.
